# Case 5 / 2018 - Acute Respiratory Failure and Cardiogenic Shock in a Patient in the First Trimester of Pregnancy with Mechanical Mitral Valve Prosthesis Implant

**DOI:** 10.5935/abc.20180205

**Published:** 2018-10

**Authors:** Walkíria Samuel Ávila, Vinícius Araújo de Freitas Chagas Caldas, Daniel Valente Batista, Paulo Sampaio Gutierrez

**Affiliations:** Instituto do Coração (InCor) HC-FMUSP, São Paulo, SP - Brazil

**Keywords:** Respiratory Insufficiency, Heart Defects, Congenital, Heart Valve Prosthesis, Shock, Cardiogenic, Pregnancy.

This case describes a 36-year-old female patient born in the state of Alagoas, and residing in the municipality of Guarulhos, state of São Paulo, Brazil, married, illiterate, admitted at the Gynecology and Obstetric Service after clinical diagnosis of upper airway infection at the 9^th^ week of the 1^st^ pregnancy.

She was followed up at the outpatient clinic specialized in congenital heart defects due to complex congenital heart disease, which included interatrial defect associated with patent ductus arteriosus and interventricular septal defect, as well as a left atrioventricular septal defect. She underwent surgery at the age of eight, consisting of atrioseptoplasty, ventriculoseptoplasty and mitral valve replacement by a mechanical prosthesis. She had paroxysmal atrial fibrillation, with a previous thromboembolic event, left hemisphere ischemic stroke, without neurological sequelae, being asymptomatic from the cardiovascular point of view, in functional class I (NYHA classification) at the last consultation in April 2018. She used only warfarin, undergoing regular follow-up of prothrombin time control/INR, having maintained it between 2-3 in the last controls.

During hospitalization in the Obstetrics Service, warfarin was replaced by enoxaparin 1mg / kg, subcutaneously, every 12 hours, and during the evolution she had atrial fibrillation with high ventricular response accompanied by dyspnea at rest and orthopnea, being subsequently referred to the Emergency Service of the Cardiology Hospital.

The physical examination at admission (May 30, 2018) showed regular overall health status, normal skin color, hydrated, anicteric, conscious, oriented, without alterations at the neurological examination. Cardiovascular examination showed regular heart rhythm, with heart rate at 115 beats per minute, holosystolic murmur, with prosthesis profile, at the superior left sternal border 2 + / 6 +, good peripheral perfusion. The respiratory system showed crackling rales on the left lung base, and mild dyspnea at rest. Gravid abdomen, with no signs of hepatic congestion. Extremities without edema, with no discomfort or pain in the calves.

The laboratory results at admission (May 30, 2018) were: hemoglobin 12.4 g / dL; leukocytes 13,050/mm³ (band cells 1%, segmented 79%, eosinophils 1%); platelets 120,000/mm³; C-reactive protein: 74.6mg / dL; Urinalysis: Leukocytes 16,000/mL, negative nitrite test, bacteria 1+/4+, Urinary culture at the hospital of origin with multisensitive *E.coli*.

The admission electrocardiogram (May 30, 2018) (Figure 1) showed sinus rhythm, heart rate of 115 bpm, indirect signs of right atrial overload.

**Figure 1:**
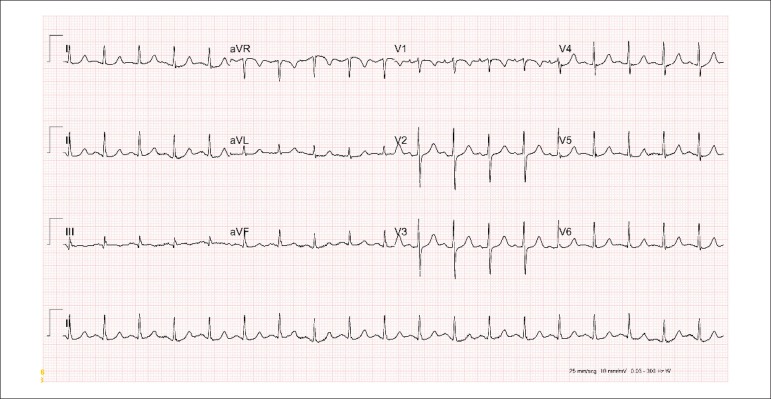
Admission ECG: sinus rhythm with indirect signs of left atrial overload and right atrial overload (Peñaloza-Tranquesi).

The admission chest x-ray (May 30, 2018) (Figure 2) disclosed indirect signs of pulmonary congestion ("cottony" infiltrate, predominantly bibasal), peri-hilar air bronchogram on the right and image compatible with mechanical prosthesis in the mitral position.

**Figure 2:**
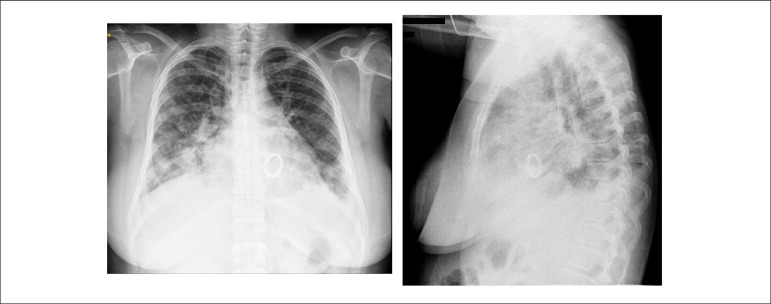
Admission chest x-ray: signs of congestion and pulmonary infection (air bronchogram).

The initial diagnosis at hospitalization was bronchopneumonia, pulmonary congestion, atrial fibrillation with high ventricular response, and a single, nine-week non-ectopic pregnancy, and she was prescribed: Ceftriaxone, Clarithromycin, Oseltamivir, Furosemide and Sotalol. The requested exams included blood culture, H1N1 virus screening, transthoracic echocardiography, and Anti-Xa factor.

During the evolution she showed signs and symptoms of pulmonary infectious disease (cough, dyspnea, leukocytosis with left shift, high PCR, with negative H1N1), and it was decided to discontinue Oseltamivir and implement empirical antibiotic therapy with Meropenem.

Compared with the patient’s last transthoracic echocardiogram, the transthoracic echocardiogram carried out on June 4, 2018 disclosed a marked increase in the mitral transvalvular gradient (maximum diastolic gradient of 39mmHg and mean of 25mmHg), in addition to an increase of pressures in the right chambers, with right ventricular systolic pressure of 75 mmHg, with no evidence of thrombi or vegetation (Table 1).

**Table 1:** Echocardiographic evolution

Echocardiographic measures	Date		
	Pre-admission	June 04, 2018	June 14, 2018
Aorta (mm)	24	28	28
Left atrium (mm)	45	55	57
Right ventricle (mm)	24	26	41
Septum (mm)	10	9	9
Posterior wall (mm)	9	10	9
Left Ventricle Diast./Syst. (mm)	53/33	40/28	40/25
LVEF (%)	Normal	Normal	Normal
Max. trans-mitral gradient (mm Hg)	16	39	45
Mean trans-mitral gradient (mm Hg)	6	25	30
Mitral prosthesis (mobility)	Good	Low	Low
Thrombus	No	No	Yes
RV Syst pressure (mm Hg)	46	75	73

Diast.: diastolic; Syst.: systolic; LVEF: left ventricular ejection fraction; RV: right ventricle.

Furosemide and metoprolol were added to the antibiotics aiming at heart rate control, in addition to anticoagulation maintenance with enoxaparin with adequate levels of Anti-Xa factor (between 0.8 and 1U/mL) with improvement of clinical status. A transesophageal echocardiogram was requested for a more adequate assessment of the valve prosthesis (June 14, 2018). This examination showed the reduction in the mobility of the mitral prosthesis components, with a high mean transvalvular gradient (30 mmHg and a hypoechogenic image occupying the central region of the atrial face of the prosthesis, compatible with a thrombus). Its measurements, even underestimated, since it was difficult to determine its full extent using the two-dimensional methodology, reached values of 0.9 x 1.3 cm, resulting in an area of 1.17 cm² (important when > 0.8 cm²) and, thus, the surgical intervention was indicated, since it was available at the service (Table 1). Given the echocardiographic diagnosis of mitral valve prosthesis thrombosis, surgical treatment of the mitral valve was indicated, despite the gestational age, due to the high risk of maternal death. Intravenous unfractionated heparin was then started in an infusion pump while awaiting the surgical procedure.

During this period, the patient developed a new picture of marked dyspnea, with marked congestion (Figure 3), tachycardia, and fever, requiring invasive ventilatory support with orotracheal intubation, and hypotension requiring vasopressor agent (noradrenaline). She went into cardiorespiratory arrest for 6 minutes, with spontaneous circulation and frank shock, requiring high doses of noradrenaline, adrenaline and vasopressin. She remained in shock and, despite the measures, presented with bradycardia and asystole and died (7h22; June 18, 2018). (Dr. *Walkíria Samuel Ávila)*

**Figure 3:**
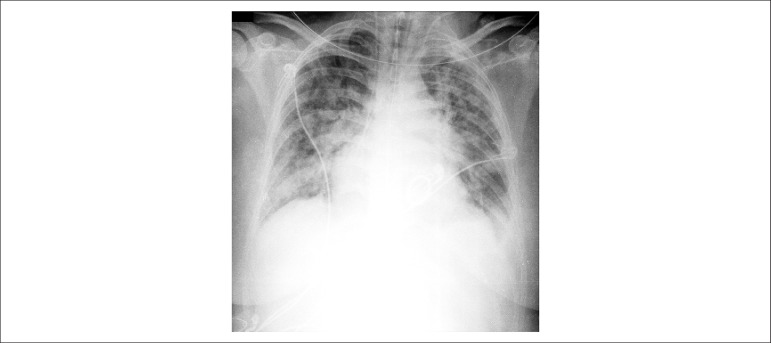
Chest X-ray showing significant pulmonary congestion.

## Clinical aspects

The case reported is of a 36-year-old pregnant woman with repaired complex congenital heart disease, with a mechanical mitral valve prosthesis implanted 28 years before, paroxysmal atrial fibrillation, and a history of thromboembolism, a triad that characterizes a high thromboembolic risk.^1^

Notwithstanding, the patient maintained the adequate anticoagulation goal (INR = 3) until the pregnancy diagnosis, when the anticoagulation regimen of warfarin was replaced by enoxaparin due to the risk of fetal warfarin syndrome, which occurs between the 6^th^ and 12^th^ weeks of gestation (characterized by nasal hypoplasia, dysplasia of the bony epiphyses, limb deformities, neurological and respiratory problems).^1^ However, there is less evidence of the erratic bioavailability and distribution of enoxaparin during pregnancy,^2^ although it constitutes a current challenge to define the best anticoagulation strategy in this population with high thromboembolic risk.

As a therapeutic option for the treatment of prosthesis thrombosis, the thrombolysis with streptokinase or alteplase, guided by serial transesophageal echocardiography, was shown to be safe and effective.^3^ However, considering the clinical situation of the patient, such as NYHA functional class IV, the need for intensive care, mechanical mitral prosthesis with a thrombus size > 0.8 cm², the surgical treatment was chosen.^4^^-^^6^^)^ Despite the established supportive care, while waiting for the previously indicated definitive surgical therapy, the patient showed clinical deterioration and died, alerting us to the potential severity of a prosthesis thrombosis picture, which requires an emergency procedure (surgical or thrombolysis), regardless of aggravating factors such as the pregnancy itself or associated infections. (Dr. Vinícius Araújo de Freitas Chagas Caldas and Dr. Daniel Valente Batista)

**Diagnostic hypotheses:** cardiogenic shock, acute pulmonary edema, thrombosis of the mechanical mitral prosthesis, systemic inflammatory response syndrome with possible pulmonary infectious focus. (Dr. Vinícius Araújo de Freitas Chagas Caldas and Dr. Daniel Valente Batista)

## Necropsy

**Figure 4:**
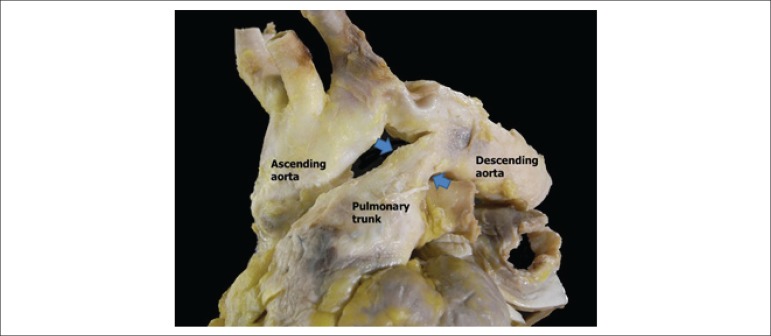
Great arteries of the heart showing patent ductus arteriosus (between the arrows).

**Figure 5:**
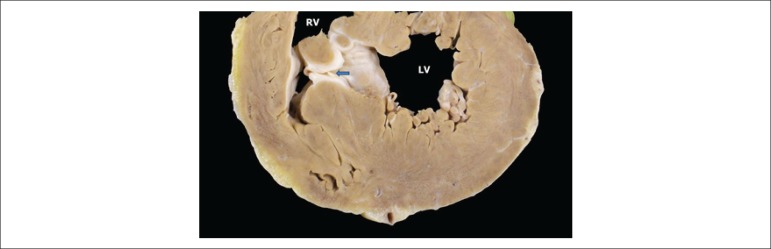
Transversal section of the heart at the region of the ventricles showing a muscular ventricular septal defect (arrow). RV- right ventricle; LV- left ventricle.

**Figure 6:**
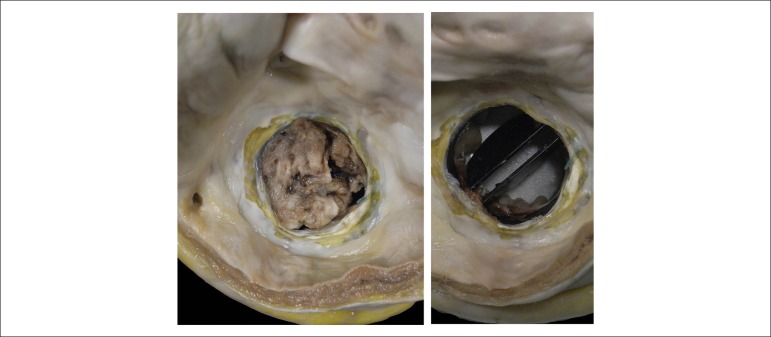
The mechanical valve prosthesis is seen from the opened left atrium. The left panel shows a massive thrombus occluding almost completely the valvar orifice. After removal of the thrombus, (right panel), it is demonstrated that the prosthesis shows adequate opening.

**Figure 7:**
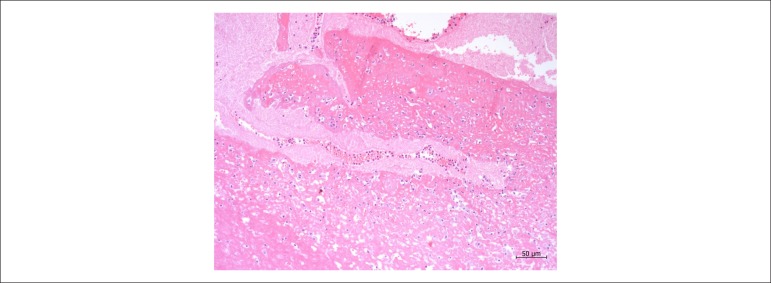
Histological section of the mass adhered to the valve prosthesis, constituted by thrombus, with fibrin and moderate amounts of inflammatory cells, without microorganisms. Hematoxylin & eosin staining; objective magnification: 10x.

**Figure 8:**
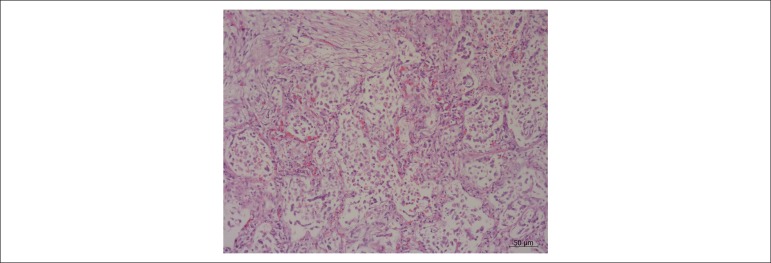
Histological section of the lung showing alveoli filled by mononuclear cells and presence of collagen. Hematoxylin & eosin staining; objective magnification: 10x.

The gravid uterus contained an apparently well-formed fetus. The mother had a mild degree of pulmonary emphysema and significant alterations in the cardiovascular system, with a patent ductus arteriosus (Figure 4) measuring 2 mm in diameter; small interventricular septal defect (Figure 5); surgical sutures in the atrial septum, possibly corresponding to the defect closure; embolism (or thrombosis) of the left subclavian vein; and mechanical valve prosthesis in the mitral position, occluded by the presence of a thrombus-like mass in the two faces (Figure 6). Microscopic study confirmed the nature of this mass, with absence of microorganisms (Figure 7). There were small infarcts in the right kidney, possibly due to embolism caused by the prosthesis thrombus, and in the subendocardial region of the left ventricle. The lungs showed many alterations, almost in their entirety, with a histopathological pattern of organizing pneumonia (Figure 8). Furthermore, demonstrating congestion, there were macrophages containing hemosiderin, but not in large numbers; and dilation of lymphatic vessels. (Paulo Sampaio Gutierrez)

**Anatomopathological diagnoses:** Congenital heart disease with interatrial defect, interventricular defect, patent ductus arteriosus, and thrombosis of the mechanical mitral valve.

**Cause of death:** Mitral valve obstruction / organizing pneumonia (Dr. Paulo Sampaio Gutierrez)

## Comment

Although the main problem that led to the death of the patient was thrombosis of the mitral valve prosthesis - emphasizing the difficulty of managing the coagulation system during pregnancy - it is worth mentioning that the lungs were also very affected, with a pattern of organizing pneumonia. It is important to emphasize that the diagnosis of “organizing pneumonia” refers to a picture that may follow not only classical bacterial pneumonia, but also several other situations, such as viral infections, exposure to toxic inhalants and others.^7^ However, marked congestion, albeit sudden, is not listed among the possible causes of this process. Therefore, in the present case, organizing pneumonia must have been due to the respiratory picture, possibly an infectious one, whether viral or bacterial, which was already present when the patient was admitted at the institution.
